# Supplementary material for: Identification of Human UDP-Glucuronosyltransferase Involved in Gypensapogenin C Glucuronidation and Species Differences
Source: Int J Mol Sci. 2023 Jan 11;24(2):1454. doi: 10.3390/ijms24021454 (PMC9865363; doi:10.3390/ijms24021454)
Supplement: Supplementary file 1 [file ijms-24-01454-s001.zip › ijms-2107344-supplementary.pdf]

Supplementary Materials

Identification of Human UDP-Glucuronosyltransferase Involved in Gypensapogenin C Glucuronidation and Species Differences

Juan Chen [1], Lin Qin [1,2], Xingdong Wu [1,2], Daopeng Tan [1,2], Yanliu Lu [1,2], Yimei Du [1,2], Di Wu [1,2], Yuqi He [1,2,*]

1 Guizhou Engineering Research Center of Industrial Key-technology for Dendrobium Nobile, Zunyi Medical University, Zunyi 563000, China

2 Joint International Research Laboratory of Ethnomedicine of Ministry of Education, Zunyi Medical University, Zunyi 563000, China

* Corresponding author: Tel.: +86-17785211152

E-mail address: yqhe2016@zmu.edu.cn

Figure S1. Glucuronidation of Gypensapogenin C at different protein concentrations.

Figure S2. Glucuronidation of Gypensapogenin C at different incubation times.

Table S1. Correlation coefficients between protein concentration and Gypensapogenin C glucuronidation.

Table S2. Correlation coefficients between incubation time and Gypensapogenin C glucuronidation.

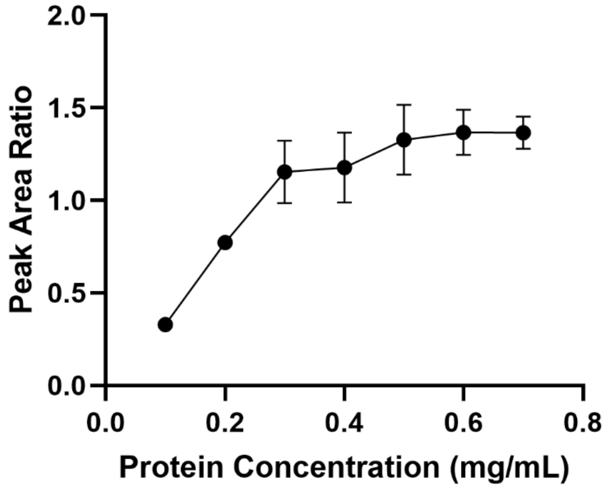

Figure S1. Glucuronidation of Gypensapogenin C at different protein concentrations. Data are from three replicate experiments and results are expressed as mean ± SD.

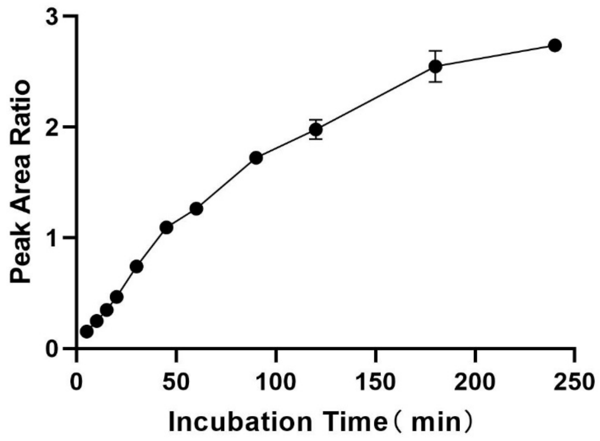

Figure S2. Glucuronidation of Gypensapogenin C at different incubation times. Data are from three replicate experiments and results are expressed as mean ± SD.

Table S1. Correlation coefficients between protein concentration and Gypensapogenin C glucuronidation.

Protein concentration rang (mg/mL)	Correlation coefficient
0.1-0.3	0.9982
0.1-0.4	0.8981
0.1-0.5	0.8831
0.1-0.6	0.8490
0.1-0.7	0.7955

Table S2. Correlation coefficients between incubation time and Gypensapogenin C glucuronidation.

Incubation time rang (min)	Correlation coefficient
5-15	0.9999
5-20	0.9979
5-30	0.9925
5-45	0.9973
5-60	0.9870
5-90	0.9831
5-120	0.9698
5-180	0.9597
5-240	0.9314
